# Development and validation of the Thai mental well-being scale

**DOI:** 10.1016/j.heliyon.2022.e09296

**Published:** 2022-04-18

**Authors:** Saran Pimthong, Charin Suwanwong, Amaraporn Surakarn, Araya Chiangkhong, Thanayot Sumalrot, Anon Khunakorncharatphong

**Affiliations:** a Behavioral Science Research Institute, Srinakharinwirot University, Bangkok, Thailand; b Independent Researcher, Bangkok, Thailand; c Graduate School, Srinakharinwirot University, Bangkok, Thailand; d Faculty of Nursing, Navamindradhiraj University, Bangkok, Thailand; e Faculty of Medicine Siriraj Hospital, Mahidol University, Bangkok, Thailand; f International Health Policy Program (IHPP), Ministry of Public Health, Nonthaburi, Thailand

**Keywords:** Mental well-being, Scale development, Validation, Thailand

## Abstract

Mental well-being is a state of positive mental health, but there is currently no valid tool for assessing mental well-being in the Thai population. The purpose of this research was to develop and validate a mental well-being scale for Thai people. Data from 2000 Thai citizens were analyzed to explore the structural components of mental well-being. Exploratory factor analysis (n = 1000) was conducted and determined a mental well-being factor structure consisting of three factors: 1) positive emotion and thinking, 2) positive relationship and 3) positive functioning. Confirmatory factor analysis of a validation group (n = 1000) identified 10 items within these three factors of mental well-being. Psychometric analyses supported internal consistency reliability, as well as convergent and discriminant validity of the scale. The findings suggest that the construction of this new mental well-being scale for Thai people is reliable and valid. This scale will be a useful tool for addressing and identifying mental health problems among the Thai population.

## 1 Introduction

Previous studies on mental well-being have centered on two distinct concepts (Ryan and Deci, 2001), namely, hedonic and eudaimonic. Hedonic well-being focuses on the personal experience of happiness and overall satisfaction with life known as subjective well-being (SWB) (Kahneman et al., 1999). Eudaimonic well-being, also known as psychological well-being, describes the mental functioning and self-awareness required to achieve one's full potential (Ryff and Singer, 2008). Many studies describe the state of well-being in terms of these two different perspectives (Giuntoli et al., 2021; Joshanloo et al., 2021; Paleari et al., 2021). However, most contemporary psychologists disagree with the distinct categorical segregation of well-being, arguing that the integration of both types is needed to achieve a flourishing and good life (Ryff et al., 2021; VanderWeele, 2017).

Mental well-being implies having positive mental health, that is, to feel good and to function well. It is a concept developed from the integration of hedonic and eudaimonic well-being (De Cates et al., 2015; Santini et al., 2020). According to the World Health Organization (WHO, 2004), positive mental health is the foundation of well-being and occurs at both the individual and community levels. The WHO defines positive mental health as a "state" by which an individual is aware of their abilities, able to deal with daily stress, and work efficiently, with support from their communities. Compared to mental well-being, "ill-being", or the presence of a mental disorder, is on the opposite spectrum (Huppert, 2014).

Currently, the most popular and widely used tool for measuring mental well-being is the 14-item Warwick-Edinburgh Mental Well-Being Scale (WEMWBS). Developed by Tennant et al. (2007), this tool is considered suitable for assessing mental well-being and has been validated in diverse population groups (Lang and Bachinger, 2017; Taggart et al., 2013; Trousselard et al., 2016) and several countries (Fung, 2019; Hoffman et al., 2019; Koushede et al., 2019; Lang and Bachinger, 2017; Santos et al., 2015; Smith et al., 2017; Trousselard et al., 2016). The scale has been modified to a shorter version (Haver et al., 2015; Koushede et al., 2019; Shah et al., 2018) that is reliable and easy to use. In addition, the WEMWBS is sensitive to changes when applied within a health promotion context (Ringdal et al., 2018; Shah et al., 2018). However, studies of mental well-being within a unique culture such as Thailand, require sociocultural consideration. A review of past literature showed that Eastern people view social relationships as part of mental well-being (Jiang and Ngai, 2020; Maulana et al., 2019; Vaingankar et al., 2021). Therefore, to accurately measure the mental well-being of the Thai population, there is a need both to develop and evaluate the reliability and validity of a new scale specific to Thai culture.

This study aimed to develop a scale to measure the mental well-being of the Thai people and to assess its psychometric performance specifically reliability and validity.

## 2 Methods

### 2.1 Participants and procedures

As recommended by Comrey and Lee (1992), a sample size of 1,000 could be excellent for factor analysis. To avoid issues related to common method variance, there should be one sample for exploratory factor analysis (EFA) and a different sample for confirmatory factor analysis (CFA) (Jordan and Troth, 2020). Therefore, our sample was divided into two groups with the first group (n = 1000) undergoing the exploratory factor analysis, whereas the second group (n = 1000) was used for confirmatory factor analysis. Finally, a total sample of 2,000 Thai people were collected.

A multistage random sampling method was conducted. In the first stage, one province was randomly selected from each region as a research site: Chiang Mai (north), Nakhon Ratchasima (northeast), Chonburi (east), Prachuap Khiri Khan (west), Phuket (south) and Bangkok (central). In the second stage, two districts were randomly selected from each province: urban and rural areas. In the third stage, Thai people from the districts were invited to participate through an online survey. The inclusion criteria for participants were as follows: (a) age more than 15 years old; (b) understand Thai language; (c) willingness to participate in the study. Non-Thai and those unable to access the internet were excluded. Most of the participants were female (59.4%). The average age was 32.80 (range: 15–89) years of age.

The research complied with all research ethics and privacy rights. The Human Research Ethics Committee, Strategic Wisdom and Research Institute of Srinakharinwirot University issued an approval letter (no. SWUEC-209/2564E). All participants read and signed the consent letter before completing any study activities. Parental consent was obtained if participants were below 18 years of age. The consent letter contained details of the research objectives, confidentiality of participants' information, and the right to withdraw from the research at any time.

### 2.2 Scale development

**Figure 1:**
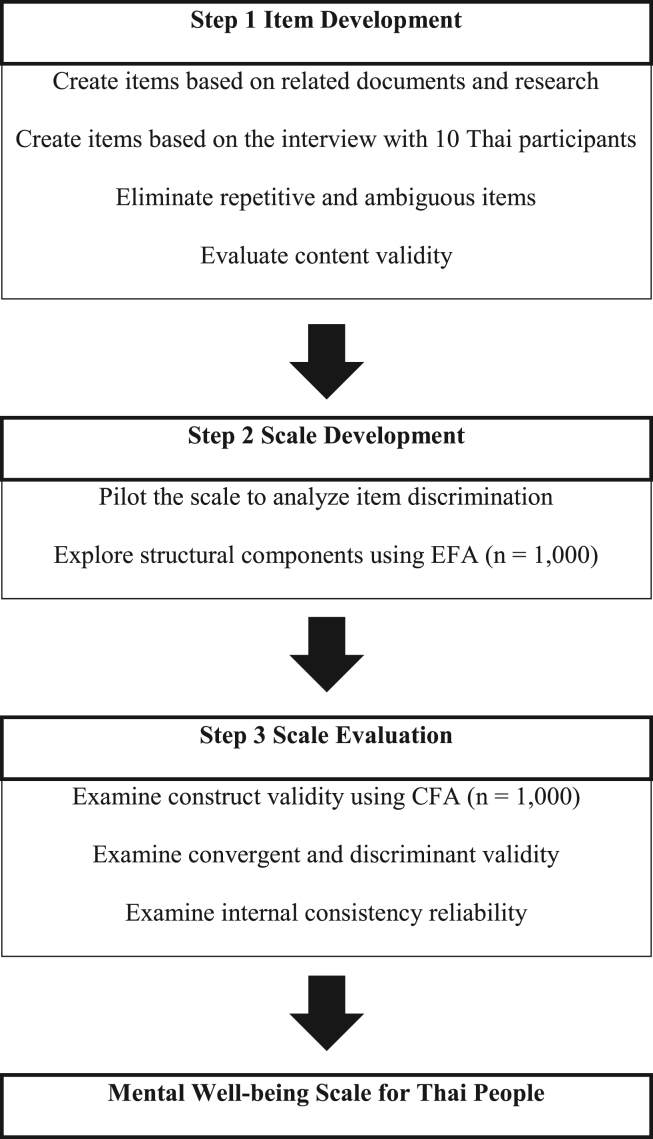
Development process of mental well-being scale.

The development of the mental well-being scale followed the process proposed by Boateng et al. (2018) as conceptualized in Figure 1.

The preliminary 40 item content of the Thai mental well-being scale was created by using deductive and inductive methods. The deductive methods involved reviewing relevant documents and previous studies (Haver et al., 2015; Nima et al., 2020; Sham et al., 2021; Tennant et al., 2007; Topp et al., 2015). The inductive method was used to extract information about the construct of the instrument. In-depth interviews with the semi-structure guideline were used to collect and generate the initial items pool. The first author conducted the interviews. A total of 10 key informants from various settings, were chosen based on purposive sampling. After screening for duplicate and ambiguous questions, 21 items remained. Content validity was then evaluated by five experts with knowledge and experience in the field of psychometric assessment, mental health and behavioral sciences. In addition, they were asked to evaluate the relevant items (−1 = no; 0 = undecided; +1 = yes). After we calculated an index of item-objective congruence (IOC), it was found that all 21 items had an index of item-objective congruence (IOC) greater than 0.50 (Rovinelli and Hambleton, 1977). All items were constructed using a 6-point Likert scale, ranging from 1 “strongly disagree” to 6 “strongly agree”. The initial scale was administered to a pilot group of 30 people to assess the quality of each item. The results showed that all items had corrected item-total correlations between 0.42-0.85, and all were greater than 0.30 (Boateng et al., 2018).

### 2.3 Data analysis

For the EFA, principal component analysis was performed using the Varimax rotation method to explore mental well-being components, considering components with eigenvalues greater than 1, items with commonalities greater than 0.40, and factor loading greater than 0.50 (Hair et al., 2014). For the CFA, the following goodness of fit indices were used: a) nonsignificant chi-square, b) root mean square error of approximation (RMSEA) less than 0.08, c) comparative fit index (CFI) greater than 0.95; d) Tucker–Lewis Index (TLI) greater than 0.95, and e) standardized root mean square residual (SRMR) less than 0.08 (Brown, 2015).

## 3 Results

### 3.1 Exploratory factor analysis

Before performing exploratory factor analysis, we confirmed analytic suitability of the variables by using the Kaiser–Meyer–Olkin Measures of Sampling Adequacy (KMO), which was equal to 0.94, and Bartlett's Test of Sphericity, which was equal to 12995.21, p < .01. Initial EFA analysis identified three factors with eigenvalues greater than 1, with a cumulative variance of 62.32%. Some items were eliminated due to their commonalities being less than 0.40 and their factor loading being less than 0.50. After repeating the EFA to reduce the number of items, 16 items within the three factors remained. The cumulative variance was 66.37% (Table 1). The first factor, “Positive Emotion and Thinking” (5 items), explained 23.35% of the variance. The second factor, “Positive Relationship" (6 items), explained 23.17% of the variance. The third factor, “Positive Functioning” (5 items), explained 19.85% of the variance.

**Table 1:** Factor loading of mental well-being (16 items).

No.	Item	Factor Loading		
		Positive Emotion and Thinking	Positive Relationship	Positive Functioning
**Positive Emotion & Thinking**				
M1	I know that everything in the future will be fine	**0.81**	0.17	0.14
M2	I feel relaxed	**0.82**	0.14	0.17
M4	I feel that there will only be good things happen in my life	**0.76**	0.29	0.27
M5	I feel that all problems will eventually go away	**0.74**	0.23	0.25
M6	I feel fresh and bright	**0.77**	0.29	0.28
**Positive Relationship**				
M7	I feel close with people around me	0.34	**0.64**	0.24
M8	I feel loved by people around me	0.32	**0.72**	0.20
M9	I feel comfort being around with my close ones	0.17	**0.80**	0.13
M10	I like spending time with my beloved ones	0.08	**0.74**	0.16
M11	I have a person I can trust	0.12	**0.79**	0.14
M12	I am satisfied with my relationships with people around me	0.28	**0.72**	0.24
**Positive Functioning**				
M13	I never run out of energy	0.34	0.18	**0.75**
M14	I am good at dealing with problems	0.41	0.18	**0.68**
M15	I am able to clearly express my opinions	0.21	0.20	**0.72**
M18	I am able to make decisions by myself	0.12	0.17	**0.82**
M19	I am interested in new things	0.11	0.20	**0.69**

### 3.2 Confirmatory factor analysis

**Figure 2:**
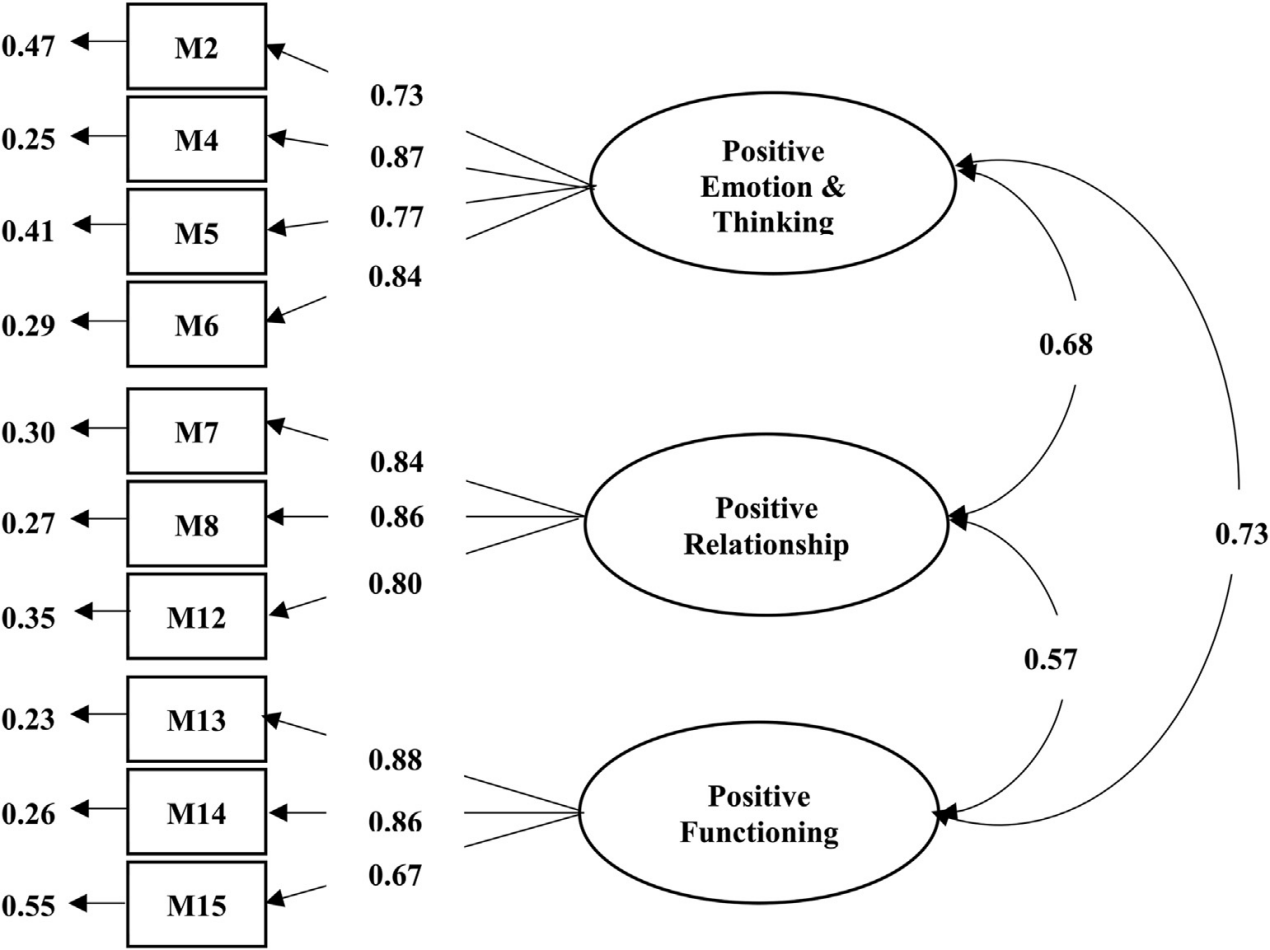
CFA of the 10-item mental well-being scale.

The initial results of the CFA suggested that the hypothesis-based model was not consistent with the empirical data. Therefore, we decided to exclude items with factor loadings less than 0.50 (Hair et al., 2014) and used the modification index (MI) by drawing lines to illustrate relationships between the items until the model had a better fit: χ^2^ = 30.05, df = 23, p > 0.05, CFI = 1.00, TLI = 1.00, RMSEA = 0.02, and SRMR = 0.01. This led to a mental well-being model with 3 factors totaling 10 items: positive emotion and thinking (4 items), positive relationship (3 items), and positive functioning (3 items), as shown in Figure 2.

Convergent validity was then examined to demonstrate the consistency of the items used in the assessment of the same subject (Hair et al., 2014). The factor loading was between 0.67-0.88, which was greater than 0.50. The average variance extracted (AVE) was between 0.65-0.70, which was greater than 0.50. The construct reliability (CR) was between 0.85-0.88, which was greater than 0.70. In aggregate, these results demonstrate sufficient convergent validity. Discriminant validity was examined to evaluate the ability to discriminate between the components of the variables (Hair et al., 2014). The AVE (0.65–0.70) was greater than the square of the correlation value between the components (the highest value was 0.53). Thus, there was evidence to support discriminant validity (Table 2).

**Table 2:** Validation results of the mental well-being scale.

No	Item	CITC	Cronbach's Alpha	AVE	CR
**Positive Emotion & Thinking**			**0.88**	**0.65**	**0.88**
M2	I feel relaxed	0.66			
M4	I feel that there will only be good things happen in my life	0.77			
M5	I feel that all problems will eventually go away	0.67			
M6	I feel fresh and bright	0.78			
**Positive Relationship**			**0.86**	**0.70**	**0.87**
M7	I feel close with people around me	0.64			
M8	I feel loved by people around me	0.67			
M12	I am satisfied with my relationships with people around me	0.63			
**Positive Functioning**			**0.75**	**0.65**	**0.85**
M13	I never run out of energy	0.64			
M14	I am good at dealing with problems	0.67			
M15	I am able to clearly express my opinions	0.53			
**Total**			**0.91**		

CITC = Corrected Item-Total Correlation, AVE = Average Variance Extracted, CR = Construct Reliability.

### 3.3 Internal consistency

Cronbach's alpha of the 10-item mental well-being scale was 0.91, indicating excellent internal consistency reliability. When considering each factor, the Cronbach's alpha values for positive emotion and thinking, positive relationship, and positive functioning were 0.88, 0.86, and 0.75, respectively. In addition, all items had corrected item-total correlations ranging from 0.53-0.78, which were greater than 0.30 (Table 2).

## 4 Discussion

The objective of this study was to develop and validate a mental well-being scale by using data collected from a nationwide sample of Thai people. The creation of our mental well-being scale adhered to the rigorous guidelines of Boateng et al. (2018). The results showed that this newly developed scale has suitable psychometric properties, specifically those of validity and reliability.

### 4.1 The Thai mental well-being scale

We reviewed both source documents and prior research and conducted interviews with a group of Thai people. Thus, our mental well-being scale was built using both deductive and inductive methods; an approach that is more comprehensive than using theoretical perspectives alone. Both methods are recommended in scale development since variable definitions are often considered the most important step in the process (Morgado et al., 2018).

Based upon the results of both the EFA and CFA, the newly developed mental well-being scale is psychometrically reliable and valid. The EFA revealed three structural components of mental well-being and the CFA confirmed these multidimensional structures. The final scale contains 10 items, consisting of 3 components: positive emotion and thinking (4 items), positive relation (3 items), and positive functioning (3 items). The results of the CFA demonstrated that the items are representative of their respective components, serving as empirical evidence for construct validity. Evidence for discriminant and convergent validity was also found. As emphasized by Boateng et al. (2018), these specific psychometric properties should be represented in newly created scales.

The first component of our scale, positive emotion and thinking, assesses positive feelings and thoughts. Those with higher scores are more positive in terms of their emotions and thoughts compared with those with lower scores. This factor is consistent with subjective well-being or the hedonic concepts of satisfaction, cheerfulness, and life assessment (Huta, 2017; Tov, 2018). Hedonic well-being is essential for individuals to maintain good physical and mental health, as well as for longevity (Chei et al., 2018; Fastame et al., 2021; Zaninotto and Steptoe, 2019). The second component, positive relationship, refers to a positive view of one's relationships with others, including the benefits from personal connections. Those with higher scores are more positive about their relationships compared with those with lower scores. Like past research (Maulana et al., 2019; Saeri et al., 2018; Tough et al., 2017), our findings suggest that connection with others is an important part of well-being. For Thai people, good relationships also include providing and receiving emotional support, both of which are crucial for a person to be psychologically healthy (Auttama et al., 2021; Sasiwongsaroj et al., 2015; Thanakwang, 2015). The third component, positive functioning, describes the resources and strengths necessary for fulfilling one's potential. Those with higher scores are more positive about these areas of functioning compared with those with lower scores. This factor is also consistent with the awareness of human potential cited to be important to psychological or eudaimonic well-being (Brandel et al., 2017). Eudaimonic well-being is essential in promoting good mental health, a flourishing life, and moving towards future goals (Joshanloo et al., 2021; Ryff, 2014, 2017).

### 4.2 Implications

The applications of this mental well-being scale are as follows: 1) The three components of the mental well-being scale include items that reflect good mental health. Thus, Thai people can use this scale for initial mental health screening; 2) The mental well-being scale contains information related to positive mental health. Researchers and government agencies can use these mental health outcome variables to develop policies and programs related to the well-being of Thai people; and 3) Items from the mental well-being scale can be used for identifying mental health problems and understanding characteristics related to mental health that may need improvement. For example, a person may possess positive emotions, but lack good relationships with others. With such information, it would be possible to develop a personalized intervention program to focus on areas that need improvement.

### 4.3 Limitations and future research

This scale included a series of items that were quite generic; therefore, its validation should be in Thai-focused studies with extension to some specific studies in South-East Asia. Consequently, the scale must be tested and adapted before implementation in other contexts. Moreover, even though the scale possesses excellent psychometric properties, it has not been validated, using other standard methods. Other forms of validation, for example, concurrent validity, predictive validity, and test-retest reliability should be examined in future studies.

## 5 Conclusions

We developed and validated a mental well-being scale for Thai people, consisting of 10 items, divided into 3 components: positive emotion and thinking (4 items), positive relationship (3 items), and positive functioning (3 items). This scale uses a 6-point Likert scale ranging from “not true at all” to “absolutely true”. Those who scored higher show better mental well-being than those with lower scores. The scale has suitable psychometric properties, specifically validity and reliability. The scale, therefore, will be useful for assessing and identifying mental health problems and as an indicator for the development of mental well-being programs for the Thai people.

## Declarations

### Author contribution statement

Saran Pimthong; Amaraporn Surakarn; Araya Chiangkhong; Thanayot Sumalrot; Anon Khunakorncharatphong: Conceived and designed the experiments; Performed the experiments; Analyzed and interpreted the data.

Charin Suwanwong: Conceived and designed the experiments; Performed the experiments; Analyzed and interpreted the data; Contributed reagents, materials, analysis tools or data; Wrote the paper.

### Funding statement

This work was supported by 10.13039/501100009061Thai Health Promotion Foundation.

### Data availability statement

Data will be made available on request.

### Declaration of interests statement

The authors declare no conflict of interest.

### Additional information

No additional information is available for this paper.
